# A Rare Case of TFE3‐Rearranged and SMARCB1‐Mutated Renal Cell Carcinoma: Diagnostic and Therapeutic Complexities

**DOI:** 10.1155/crom/6909249

**Published:** 2026-02-24

**Authors:** Paul J. Pecorin, Nyembezi Dhliwayo, Trevor Christ, Thomas Westbrook

**Affiliations:** ^1^ Department of Medicine, Rush University Medical Center, Chicago, Illinois, USA, rush.edu; ^2^ Division of Hematology, Medical Oncology and Cell Therapy, Department of Medicine, Rush University Medical Center, Chicago, Illinois, USA, rush.edu

## Abstract

Renal cell carcinoma (RCC) represents the majority of kidney malignancies, with clear cell RCC being the most common subtype. However, recent advances in tumor genomics have refined RCC classification, highlighting rare molecular subtypes such as TFE3‐rearranged and SMARCB1‐deficient RCCs. These entities pose unique diagnostic and therapeutic challenges due to their aggressive nature and limited treatment data. We present a rare case of metastatic RCC with concurrent TFE3 translocation and SMARCB1 mutation in a 39‐year‐old patient. The patient underwent multiple systemic therapies, including immune checkpoint inhibitors (ICIs) and tyrosine kinase inhibitors (TKIs), alongside surgical and radiation interventions. Despite disease progression, a combination of lenvatinib and pembrolizumab has shown recent stabilization. This case underscores the importance of genomic profiling in identifying rare RCC subtypes and guiding treatment decisions. Given the aggressive course and lack of established guidelines, further research is needed to optimize therapeutic strategies for these molecularly defined RCC variants.

## 1. Introduction

Renal cell carcinoma (RCC) makes up a large majority of kidney malignancies, up to 90%. RCC can be further classified into many subgroups, with clear cell RCC being the most common [1].

The World Health Organization recently updated kidney cancer classification in 2022. One subgroup of nonclear cell RCC is the molecularly defined renal carcinomas, informed by recent advances in tumor genomics. This category includes transcription factor binding to IGHM enhancer 3 (TFE3) rearranged and transcription factor EB (TFEB) altered RCCs, formerly classified under the microphthalmia‐associated transcription factor (MITF) family of translocation‐associated RCC. Additionally, SWI/SNF‐related, matrix‐associated, actin‐dependent regulator of chromatin subfamily B member 1 (SMARCB1)–deficient medullary‐like RCC and SMARCB1‐deficient undifferentiated RCC are now grouped within the molecularly defined class of renal carcinoma [2].

TFE3 rearranged RCC is characterized by gene fusions involving the TFE3 gene on Chromosome Xp11.2. Frequent fusion partners for TFE3 include ASPSCR1, PRCC, and SFPQ among others. ASPCR‐1‐TFE3 fusion specifically is associated with aggressive features [3]. TFE3‐rearranged RCC is more common in children and accounts for up to 40% of pediatric RCCs, whereas accounting for only 4% of RCCs in adults [4]. The most characteristic histologic pattern is papillary architecture with clear cells and psammoma bodies. When using histology alone, these tumors can often mimic other more common forms of RCC. TFE3‐rearranged RCC can be more precisely identified using TFE3 break‐apart fluorescence in situ hybridization (FISH) assays and immunohistochemical (IHC) staining, which demonstrate TFE3 overexpression [3].

SMARCB1‐deficient RCC is another molecularly defined RCC. It is most commonly associated with renal medullary carcinoma (RMC), which primarily affects patients with sickle cell trait or other hemoglobinopathies. Although commonly seen in RMC, loss of SMARCB1 can also be seen in unclassified RCC and de‐differentiated RCC [3]. Histopathologically, these tumors exhibit high‐grade, infiltrative growth patterns and often have rhabdoid features. Diagnosis can be confirmed with immunohistochemistry demonstrating loss of SMARCB1 or FISH confirmation of genetic alterations at the SMARCB1 locus [5].

Management of localized disease is similar to clear cell RCC, either by radical or partial nephrectomy, ablative techniques, embolization and/or stereotactic body radiation (SBRT) [6]. However, these molecular subtypes are rare and often portend highly aggressive disease with metastatic potential, primarily including peritoneal nodal metastatic involvement, pulmonary, hepatic, and adrenal involvement. Furthermore, the role of immunotherapy as well as targeted therapies is not fully described for these pathologic variants due to the paucity of data.

In this report, we present a case of metastatic nonclear cell RCC with concurrent TFE3 translocation and SMARCB1 mutation who has undergone treatment with ipilimumab plus nivolumab, nivolumab plus cabozantinib, and now lenvatinib plus pembrolizumab in addition to numerous localized therapies. Despite significant advances in RCC classification and genomic profiling, the concurrent presence of TFE3‐rearranged and SMARCB1‐deficient RCC remains an extremely rare and poorly understood entity. This case underscores the diagnostic and therapeutic challenges posed by such tumors.

## 2. Case

A 39‐year‐old White, non‐Hispanic female presented to the emergency room with crampy right upper quadrant pain ongoing for about 36 h prior to her presentation. She has a past medical history of basal cell carcinoma of the chin, and a family history of breast cancer, basal cell carcinoma, and thyroid cancer in her mother as well as prostate cancer and chronic myeloid leukemia in her father. Laboratory workup and right upper quadrant ultrasound were unremarkable; however, computed tomography of the abdomen and pelvis showed a hypodense structure in the left kidney measuring 2.7 × 3.0 x × 3.1 cm. This was most consistent with a complicated cyst, but a solid tumor could not be ruled out. These findings were presumed incidental, and she was discharged with improved pain.

**Figure 1:**
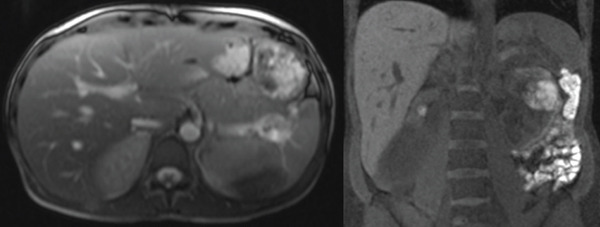
MRI showing an exophytic 6.2 × 4.2 cm lesion in the interpolar region of the left kidney later determined to be RCC. Additionally, multiple T2 hyperintense liver lesions, measuring up to 1.7 cm, can be appreciated.

She subsequently presented approximately 8 months later with 8/10 left flank pain, worsening over the preceding 72 h. At this time, she was pregnant at an estimated gestational age of 32 weeks and 1 day. Renal ultrasound showed a 5.9‐cm hyperechoic lesion in the left kidney with significant vascularity and a 9.6‐cm heterogenous hyperechoic soft tissue collection along the renal cortex. This was presumed to represent a renal angiomyolipoma with hemorrhagic rupture. CT of the abdomen and pelvis the following day showed a 7.0 × 4.9 × 8.8‐cm hematoma with a 3.0 × 2.6‐cm exophytic focus that could not be fully characterized. Magnetic resonance imaging (MRI) renal protocol further characterized the lesion as a 6.1 × 4.2‐cm lesion in the interpolar region of the left kidney favored to represent hemorrhage within a lipid‐poor angiomyolipoma with a stable hematoma (Figure 1). Although MRI was subsequently obtained, CT imaging was performed due to concern for acute hemorrhage and need for rapid evaluation. Given presumed angiomyolipoma and increased bleeding risk, induction of labor and renal artery embolization with total left nephrectomy the following day was planned. All three procedures were completed without major complication.

Pathology of left nephrectomy was thought to be consistent with high grade Type 2 papillary RCC and measured 9.5 × 6.5 × 6.0 cm with 60% of tumor consisting of hemorrhage. All tumor margins were negative for carcinoma.

Surveillance MRI abdomen and kidney showed at least four new hepatic lesions highly suspicious for metastases. She had biopsy of a liver lesion consistent with metastatic RCC. She then started on ipilimumab plus nivolumab. After 12 weeks of therapy, repeat staging scans showed further increase in size of hepatic lesions as well as a new caudate liver lesion. This was favored to be pseudo‐progression, and immune checkpoint inhibition was continued with nivolumab.

**Figure 2:**
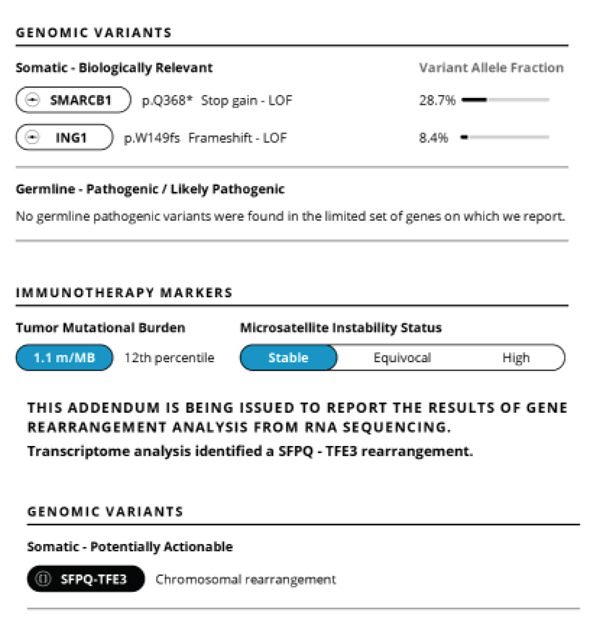
Neogenomic sequencing via Tempus^(c)^ IHC showing SMARCB1 translocation stop gain loss of function (with 28.7% variable allelic frequency), and concomitant TFE3 chromosomal rearrangement. PDL‐1 assessment with 95% tumor proportion score (combined positive score 97).

Initial assessment with genomic sequencing was done at the time with the Boston Gene Tumor Genomic Sequencing(c) assay, with findings of an SFPQ/TFE3 fusion rearrangement as well as a SMARCB1 stop gain loss of function mutation. The tumor mutational burden was low, with microsatellite stability. This was re‐demonstrated on another genomic sequencing assay by liquid biopsy via Tempus^(c)^ genomic sequencing assay (Figure 2).

On follow up imaging, she was found to have nodal metastases in the para‐aortic lymph nodes and lytic lesions in the left transverse process of L3 and inferior aspect of the sacrum. She was treated with cabozatinib and nivolumab, with subsequent imaging showing disease response.

She then underwent left hemihepatectomy, cluster resection of several metastases, and microwave ablation of eight hepatic metastases without complication. Surgical pathology of all resected metastases showed metastatic RCC. Due to enlargement of bony and nodal lesions, she received radiation therapy to her sacrum and para‐aortic lymph nodes.

**Figure 3:**
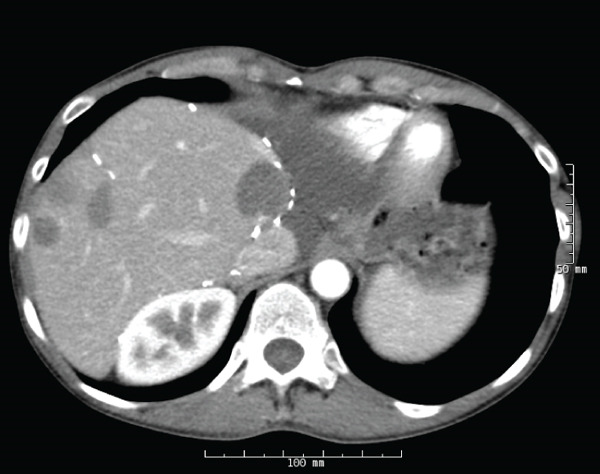
Surveillance CT abdomen and pelvis showing progression of hepatic metastases including a 1.5 × 1.4 cm subcapsular Segment VI lesion.

**Figure 4:**
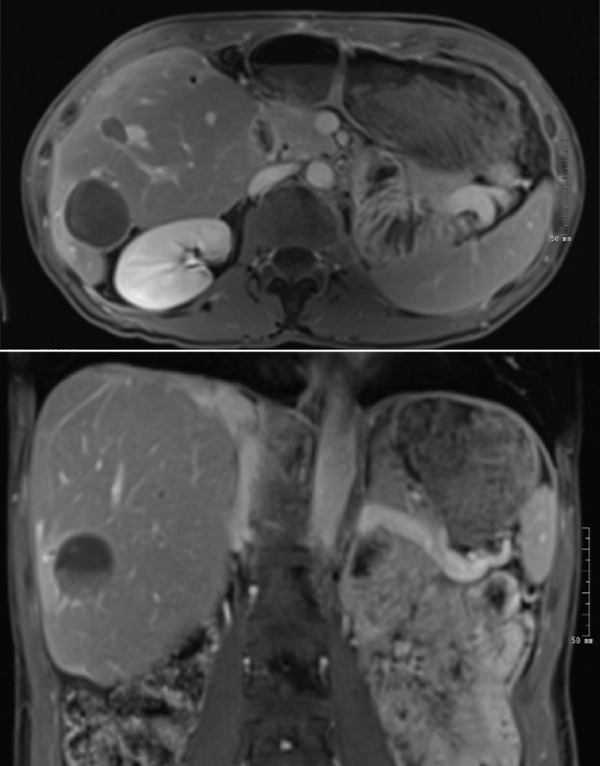
MRI abdomen with multiple foci of artifact throughout the liver consistent with ablation posttreatment changes, and interval decreased size in hepatic foci suggesting treatment response. There is no evidence of disease progression.

Scans remained stable for several months; however, eventually imaging did show progression of remaining hepatic lesions as well as a right adnexal mass. She underwent microwave ablation of hepatic lesions and had salpingo‐oophorectomy for the right adnexal lesion. The pathology of the lesion confirmed RCC metastasis. Lesions treated with microwave ablation had resolved following therapy; however, repeat imaging showed progression of disease in her liver as well as new L2 lytic lesions (Figure 3). She was then switched to lenvatinib and pembrolizumab. Staging scans including MRI abdomen and pelvis as well as CT chest were completed 1 month after starting lenvatinib and pembrolizumab. There was an interval decrease in the size of at least one liver lesion, decreasing from 1.3 × 1.2 to 1.1 × 0.9 cm, as well as a decrease in the size of the left adnexal lesion from 3.2 × 2.0 to 2.5 × 1.5 cm as compared with staging scans 3 months prior indicating mild response (Figure 4). Osseous lesions remained stable.

## 3. Discussion

RCC encompasses a heterogenous group of malignancies with distinct clinical and genomic characteristics. Nonclear cell RCC constitutes a minority of these cases; however, this class does include molecularly defined subtypes such as TFE3‐rearranged and SMARCB1‐deficient RCCs. These rare subtypes are highlighted in the 2022 WHO classifications and reflect advances in tumor genomics that are refining diagnostic criteria, and identification of molecular subtypes provides promise for directed therapies in the future [1].

In this case, we describe a unique presentation of metastatic RCC with concurrent TFE3 translocation and SMARCB1 mutation. This, in addition to a case in which these co‐existing mutations were seen in a patient with end stage renal disease who developed RCC with rhabdoid features, is an extraordinary case and portends highly aggressive disease [7]. The identification of an SFPQ/TFE3 fusion and SMARCB1 loss of function mutation underscores the role of advanced genomic profiling in refining diagnosis and guiding therapy [1]. This case contributes to the growing body of literature detailing rare molecular subtypes.

Molecularly defined RCC is associated with aggressive clinical courses as seen in this patient. Management of these entities remains challenging due to significant lack of data [2, 6]. The difficulty in management is highlighted in this case through short‐lived treatment courses including dual immune checkpoint inhibitors (ICIs) (ipilimumab and nivolumab) as well as combination VEGF‐directed tyrosine kinase inhibitor (TKI) and checkpoint inhibitor (cabozatinib and nivolumab). Combination of ICIs as well as TKI/ICI combinations have shown promise in the management of RCC; however, the paucity of data on molecularly defined RCC subtypes makes this difficult to generalize to our patient with TFE3‐rearranged and SMARCB1‐deficient RCC [6, 8, 9]. Lenvatinib, an anti‐angiogenic TKI, in combination with pembrolizumab, an anti–PD‐1 antibody, reflects recent evidence supporting this regimen in advanced RCC; although, further studies are required to further validate its efficacy in molecularly defined subtypes [10].

This case highlights the diagnostic and therapeutic challenges associated with TFE3‐rearranged and SMARCB1‐mutated RCC. Multimodal treatment, including ICIs, TKIs, surgery, and radiation, remains central to management, although durable responses are rare. The difficult decision‐making involving treatment of these patients is highlighted in the decision to rechallenge the patient with immune checkpoint inhibition following progression on combination ipilimumab and nivolumab. Although rechallenge with anti–PD‐1/PD‐L1 therapy after progression has not demonstrated benefit in clear cell RCC—as shown in CONTACT‐03 and TiNivo‐2, which both failed to show improved outcomes with continued ICI therapy and instead highlighted increased toxicity [11, 12]—these studies focused exclusively on ccRCC populations. To date, there is no definitive evidence regarding the efficacy or futility of ICI rechallenge in nccRCC, particularly in molecular subtypes such as TFE3‐rearranged and SMARCB1‐deficient RCC. Given this lack of data, the treating team considered it reasonable, though cautious, to continue immunotherapy at the time, especially as the patient strongly preferred to remain on an ICI‐based regimen, hoping for a durable response. Although this approach may not be routinely recommended, particularly considering emerging evidence in ccRCC, it reflects a patient‐centered decision made in the context of limited therapeutic alternatives and evolving data. Further investigation into the unique biology of these molecular subtypes is imperative to develop more effective and personalized treatment strategies.

## Funding

No funding was received for this case manuscript.

## Consent

Consent to write and publish this case report was obtained in writing from the patient. A written consent form has been retained by the primary author.

## Conflicts of Interest

The authors declare no conflicts of interest.

## Data Availability

The data supporting the findings of this case report are contained within the article. Additional details may be made available by the corresponding author upon reasonable request, subject to patient privacy considerations.
